# Can neutrophil-to-lymphocyte ratio predict cardiac involvement in kawasaki disease?

**DOI:** 10.1186/1546-0096-12-S1-P348

**Published:** 2014-09-17

**Authors:** Özge Altuğ Gücenmez, Balahan Makay, Mustafa Kır, Nurettin Ünal, Erbil Ünsal

**Affiliations:** 1 Pediatric Rheumatology, Dokuz Eylül University Hospital, İzmir, Turkey; 2 Department of Pediatric Cardiology, Dokuz Eylül University Hospital, İzmir, Turkey

## Introduction

Kawasaki disease (KD) is an acute self-limited vasculitis, in which 1/3 of patients develop coronary artery lesions (CAL) if untreated. Although studies have explored potential biomarkers to predict patients with KD who are at risk of CAL, no useful single marker exists. The blood neutrophil to lymphocyte ratio (NLR) is identified as a potentially useful marker of clinical outcome in inflammatory diseases.

## Objectives

To evaluate NLR in patients with KD and to investigate the relationship with coronary artery involvement.

## Methods

The hospital charts of patients who were diagnosed as KD in a single center between 2004 – 2014 were analyzed retrospectively. Demographic, clinical, and laboratory data were assessed.

## Results

Sixty KD patients were evaluated. Male:female ratio was 1.8:1. The mean age at onset of disease was 45±29 months (min: 3 max: 120 months). The mean duration of disease at the time of diagnosis was 8.2±4.6 days. The frequencies of the criteria for KD in all patients were as follows: fever more than 5 days 97%, oral mucosa changes 83%, conjunctivitis 78%, rash 73%, lymphadenopathy 51%, and extremity changes 60%. Twenty three patients (38%) had CAL. The frequencies of the criteria for KD were similar in patients with and without CAL. The duration of fever and platelet counts on admission in patients with CAL were significantly higher (p=0.004 and p=0.007, respectively) and age at onset was significantly younger (p=0.05) than the patients without CAL. The blood neutrophil to lymphocyte ratio was similar between patients with and without CAL (p=0.82). Besides; the mean platelet volume, leukocyte count, hemoglobin level, serum sodium, serum albumin and C-reactive protein level were insignificant between groups (p=0.79, p=0.16, p=0.18, p=0.51, p=0.86 and p=0.26, respectively).

## Conclusion

The significantly increased platelet count and longer fever duration along with younger age in KD patients with CAL are in agreement with those in previous literature. The results of this study suggest that blood neutrophil to lymphocyte ratio cannot predict CAL.

## Disclosure of interest

None declared.

